# A Comparison of the Physicochemical Properties and Sensory Attributes of Ricotta Cheeses Purchased from Retail Outlets in Poland

**DOI:** 10.3390/foods14081413

**Published:** 2025-04-19

**Authors:** Iwona Chwastowska-Siwiecka, Agnieszka Kaca, Jan Miciński

**Affiliations:** 1 Department of Sheep and Goat Breeding, Faculty of Animal Bioengineering, University of Warmia and Mazury in Olsztyn, Oczapowskiego 2, 10-719 Olsztyn, Poland; 2 Department of Commodity Science and Animal Raw Material Processing, Faculty of Animal Bioengineering, University of Warmia and Mazury in Olsztyn, Oczapowskiego 2, 10-719 Olsztyn, Poland; a.kaca@interia.pl

**Keywords:** ricotta cheese, physicochemical properties, color profile, sensory quality

## Abstract

The aim of this study was to compare selected physicochemical properties and sensory attributes of ricotta cheeses supplied by different producers and purchased from retail outlets in Poland. The experiment was performed on 40 fresh, unripened ricotta cheeses purchased from hypermarkets in the city of Olsztyn, Poland. The cheeses were supplied by four producers. To preserve the producers’ anonymity, the cheeses were divided into four experimental groups marked with the letters A, B, C, and D. Each group consisted of 10 cheeses supplied by the same producer. Immediately after purchase, the cheeses were transported to a laboratory for quantitative and qualitative analyses to determine their moisture contents, active and titratable acidity, shear force, color parameters (L*, a*, b*), chroma (C*), hue angles (h°), whiteness indexes (WIs), yellowness indexes (YIs), and sensory quality. The analyses revealed that the cheeses supplied by producers C and D were characterized by the highest moisture contents and the lowest titratable acidity and shear force values. The ricottas supplied by producer A were characterized by the highest values for lightness on the surface, whereas the group B cheeses were characterized by the highest contribution of redness and yellowness, as well as the highest color saturation (chroma). The contributions of redness and yellowness, chroma, and YI values were highest at the cross-sections of the group B cheeses. The cheeses supplied by producer D were characterized by visible spaces between grains, cracks, and a brittle, crumbly consistency, and they received the lowest scores for appearance at the cross-section for structure and consistency.

## 1. Introduction

Whey cheeses are a unique and underappreciated group of dairy products. This category of products includes hard, semi-hard, and soft cheeses made from proteins precipitated from gently heated whey or whole milk [1,2]. Ricotta is unripened, soft cheese that is highly popular in Greece and originated from Italy, especially in the south, but it is also popular in the Mediterranean area and internationally. It is traditionally made from whey left over from the production of mozzarella [3,4,5,6]. Ricotta is probably the oldest and best-known type of whey cheese, which has gained considerable popularity in the United States and Canada in recent decades [4]. Ricotta, especially goat’s milk ricotta, is a typical Italian cheese made from thermally coagulated whey proteins according to traditional recipes [7]. Ricotta can be produced from cow, buffalo, sheep, and goat milk whey, as well as blends of whey and milk or sweet cream [8,9]. Ricotta is produced by heating and acidifying whey to induce protein denaturation and aggregation, and the precipitated coagulant is separated, cooled, and packaged [3]. Most ricottas are produced with the addition of table salt, which is applied before acidification or added to the separated coagulant [10]. The production yield is estimated to be 4–6%, and ricotta is considered a fresh cheese with a short shelf life [11]. Ricotta and other whey cheeses are regarded as artisanal products due to the complexity of the precipitation process and critical texture and taste requirements. Milk for cheesemaking is acidified to a pH of 5.9–6.0 using a starter culture, acid whey powder, or acetic, citric, tartaric, lactic, phosphoric, or hydrochloric acids, with the addition of fruit juice (grape or lemon) or gluconolactone (GDL) [12,13,14]. Acidified milk is steam-heated, and curd particles float on the surface. Proper floatation is required for the recovery and optimal curd texture of traditional ricotta cheese [15]. Italian-type whey cheeses can be made from whole milk (with a fat content higher than 11%), semi-skimmed milk (with a fat content of 6–11%), and skim milk or whey (with a fat content below 1%) [14]. Ricotta is generally produced from whey with the addition of whole or skim milk or skim milk powder (5–20%) [16].

The production of ricotta has increased in recent years due to the growing demand for healthy and low-calorie foods [17]. The quality of ricotta is significantly influenced by animal nutrition and genetic factors as well as the physicochemical parameters of raw materials that affect the nutritional value of the final product [18,19,20]. Agabriel et al. [21], Ferlay et al. [22], and Romanzin et al. [23] demonstrated that the quality of the milk and the resulting cheese is also influenced by the botanical composition of pastures and the diet of dairy cows. At the same time, the appearance, consistency, and taste (more or less intense) of cheese are the key factors that contribute to a product’s attractiveness and appeal to consumers [4]. The whey proteins present in ricotta cheese have a higher biological value than whole chicken egg albumen or casein in tvorog. Whey proteins are the most biologically valuable food proteins; they are highly available and rich in all essential amino acids. Whey proteins also provide numerous health benefits by exerting anticarcinogenic and immunomodulatory effects. The high content of sulfur-containing amino acids significantly contributes to the nutritional value and thermal sensitivity of whey proteins [24].

Fresh, unripened ricotta is characterized by a high moisture content (70–80%) and a low calorific value of 100–135 kcal per 100 g of the product [25,26]. According to Surażyński et al. [27], the calcium content of ricotta exceeds that of tvorog and is similar to that noted in rennet cheeses. Ricotta also has a highly favorable calcium-to-phosphorus ratio. Ricotta is consumed around the world as part of various diets because it is a rich source of protein (8–10%), low in fat (10–25%) and salt (0.3–0.5%), and contains other ingredients that enhance its high nutritional value [16,28]. The variability in the concentrations of nutritional components is also influenced by the type of whey used to make ricotta (cow, sheep, goat etc.). In terms of sensory quality, whey cheeses can have a pure white, snow-white, or creamy to light gray color. They should have a brittle, creamy, delicate, and pasty consistency, with a cohesive and grainy structure. Ricotta has a characteristic fresh flavor which is determined by the type of ingredients used in the production processes. Ricotta made from whole or semi-skimmed milk has a delicate, milky, slightly sweet, and caramel flavor. Some varieties have a slightly acidic aftertaste with a mild creamy aroma [3,5,6,14,16]. Lactose contributes to the slightly sweet taste of ricotta [26]. Ricotta produced from whey or skim milk should have a slightly sweet or even a bland or neutral taste. The presence of a pungent, barnyard, or rancid aftertaste is undesirable, and it could point to an inappropriate choice of ingredients [16]. According to Obrusiewicz [1], ricotta should be moist, soft, and firm, and it should not spring back when pressed. Cheeses are composed of many interconnected grains with small holes in between. Some types of ricotta have a granular structure that gives a melting sensation in the mouth. Ricotta made from semi-skimmed milk can only have an excessively grainy and moist consistency with an undesirable, brittle, and unstable structure.

In Poland, ricotta is less popular than ripened cheeses and tvorogs, which could be attributed to low levels of consumer awareness about the nutritional value, sensory quality, and applicability of ricotta during meal preparation at home. In addition, ricotta cheeses available in retail differ considerably in composition, net weight, moisture content, salt content, acidity, brittleness, hardness, color parameters, and sensory quality [2].

The aim of this study was to compare the selected physicochemical properties and sensory attributes in ricotta cheeses supplied by different producers and purchased from retail outlets in Poland.

## 2. Materials and Methods

The experiment was performed on 40 fresh unripened ricotta cheeses purchased from hypermarkets in the city of Olsztyn (voivodeship of Warmia and Mazury, Poland). The cheeses were supplied by four different producers. The ingredient composition and nutritional value of the analyzed cheeses, declared by the producers, are presented in Table 1. The purchased products differed in shape and packaging. Cheeses supplied by two producers had a cylindrical shape (diameter: 8.5 cm; height: 5 cm); cheeses produced by one manufacturer were supplied in the form of blocks (length: 10 cm; width: 7 cm; height: 4 cm); and cheeses supplied by one producer had the shape of a truncated cone (base diameter: 10 cm; top diameter: 7.5 cm; height: 4.5 cm). Three groups of ricottas were vacuum-packaged in PE film, and one group of ricottas was packaged in PP tubs with lids. The net weight of the ricotta samples ranged from 220 to 250 g. The cheeses supplied by four producers were divided into four corresponding experimental groups marked with the letters A, B, C, and D. Each group consisted of 10 ricotta samples.

Immediately after purchase, the cheeses were transported in a self-powered portable isothermal container at a temperature of 4 ± 1 °C to the laboratory of the Department of Commodity Science and Animal Raw Material Processing of the University of Warmia and Mazury in Olsztyn for quantitative and qualitative analyses. The analyses were conducted before the expiration dates indicated on the labels. To prepare the samples for laboratory analyses, ricotta cheeses were ground to a uniform consistency in a mortar and thoroughly mixed.

### 2.1. Analytical Methods

The moisture content of the examined cheeses was determined by drying in the presence of sand at a temperature of 130 °C according to Standard PN-A-86232:1973 [29]. The percentage of water (X) was calculated using the following formula (Equation (1)):

(1) $$X=\frac{\left(a-b\right)\times 100}{\left(a-c\right)}$$

where a is the weight of the vessel containing sand, a glass rod, and cheese before drying (g), b is the weight of the vessel containing sand, a glass rod, and cheese after drying (g), and c is the weight of the vessel containing sand and a glass rod (g).

The pH levels of ricottas were measured in cheese samples emulsified in water according to Standard PN-A-86232:1973 [29]. For this purpose, 10-g cheese samples were weighed with an accuracy of 0.01 g and placed in a mortar. The samples were thoroughly ground by adding small portions of distilled water (10 cm^3^) with a temperature of 40 °C until a smooth emulsion was obtained. The resulting emulsion was brought to a temperature of 20 °C and transferred to a pH meter probe, and the pH reading was recorded to the nearest 0.05 pH unit. The analysis was conducted using a 340i pH meter equipped with a TFK 150/E temperature sensor (WTW Wissenschaftlich-Technische Werkstätten, Weilheim, Germany) and a Hamilton Double Pore combination pH electrode (Bonaduz, Switzerland). The pH meter was calibrated with buffers of known pH levels prior to measurements.

Titratable acidity was determined according to Standard PN-A-86232:1973 [29] by titrating an aqueous cheese suspension with a solution of sodium hydroxide in the presence of phenolphthalein. For this purpose, cheese samples of 5 g each were weighed with an accuracy of 0.01 g, placed in a mortar, and thoroughly ground by adding small portions of distilled water (50 cm^3^) with a temperature of 40 °C. The resulting emulsion was combined with 2 cm^3^ of phenolphthalein and titrated with a solution of sodium hydroxide until the appearance of a faint pink color that persisted for 30 s. Cheese acidity was calculated in Soxhlet-Henkel (X) degrees (°SH) using the following formula (Equation (2)):

(2) X=a×20,

where a is the volume of 0.25 N sodium hydroxide solution used in titration (cm^3^).

The shear force of the ricotta was determined according to the Warner-Bratzler method. Three cylindrical samples with a diameter of 1.27 cm and a height of 2 cm were cut out from the cheeses across the grain. The specimens were placed in an Instron 5542 machine (Canton, MA, USA) equipped with a 500 N head moving at a speed of 100 mm/min and were cut with a knife with a triangular notch. The maximum shear force required to cut the specimens was recorded.

**Figure 1:**
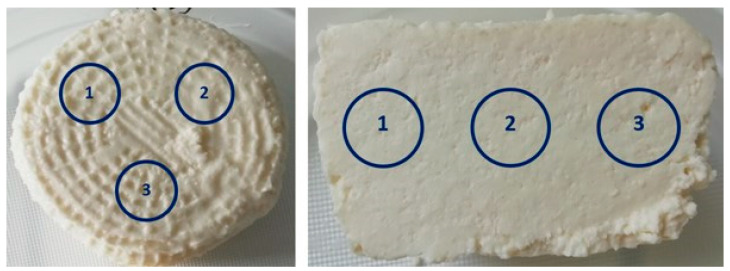
Color measurement points on the surface and at the cross-section of ricotta cheeses (source: photograph by I. Chwastowska-Siwiecka).

The color of the ricotta cheeses was determined with the use of parameters L*, a*, and b* in the CIELAB color space [30]. Color parameters were determined by measuring spectral reflectance with the HunterLab MiniScan XE Plus instrument (Hunter Associates Laboratory Inc., Reston, VA, USA). The color of the cheese samples was measured directly on the surface and at the cross-section of cheese samples. Each measurement was conducted in triplicate and at the same points (Figure 1) with a D65 standard illuminant, 10° observer, and aperture diameter of 2.54 cm. The measurements were performed immediately after cheese samples with a temperature of 4 ± 1 °C had been removed from unit packaging. The instrument was calibrated using black and white tiles before each series of measurements.

The mean values of parameters L*, a*, and b* were used to calculate the hue angle (h°) (Equation (3)), chroma (C*) (Equation (4)), the whiteness index (WI) (Equation (5)) according to the formula proposed by Rodriguez-Aguilera et al. [31], and the yellowness index (YI) (Equation (6)) according to the formula proposed by Rufián-Henares et al. [32]:

(3) $$h^{{}^{\circ}}=\tan^{-1}\frac{b^{*}}{a^{*}}$$

(4) $${\;C}^{*}=\sqrt{{\left(a\right)}^{2\;}+{\left(b\right)}^{2}},$$

(5) $$\mathrm{WI}=100-\sqrt{{\left(100-L\right)}^{2}}+a^{2}+b^{2},$$

(6) $$\mathrm{YI}=142.86\frac{b^{*}}{L^{*}},$$

The sensory analysis of the ricotta cheeses was conducted on a five-point scale according to Standard ISO 4121:2003 [33]. The following quality attributes were evaluated: color, appearance at the cross-section, structure, consistency, taste, and aroma. Each attribute was graded on a five-point scale, where 1 point denoted extremely poor quality, 2 points was unsatisfactory quality, 3 points was satisfactory quality, 4 points was high quality, and 5 points was excellent quality. The cheese samples were assessed by six panelists with validated sensory acuity (ISO 8586:2023) [34]. The samples were lit uniformly from all directions with the use of fluorescent lamps (500 lx) that simulated natural daylight and were installed at a height of about 1 m above the table. Sensory analyses were conducted at a relative humidity minimum of 60% and a temperature of 21 °C. The ricotta cheeses were evaluated based on the reference standards for the sensory attributes of fresh, unripened cheeses [33,35,36,37] and the recommendations formulated by Panfil-Kuncewicz et al. [38].

### 2.2. Statistical Analysis

The results were processed statistically by calculating the arithmetic mean ($\bar{x}$) and standard deviation (±SD). The significance of differences between the mean values of the analyzed attributes in each experimental group was determined by one-way analysis of variance (ANOVA) and Duncan’s test at *p* ≤ 0.05 and *p* ≤ 0.01. Statistical analyses were conducted in the Statistica v. 13.3 [39] program (TIBCO Software Inc., Palo Alto, CA, USA).

## 3. Results

### 3.1. Physicochemical Properties of Ricotta Cheeses

The data presented in Table 2 indicate that the analyzed cheese groups differed (*p* ≤ 0.01) in terms of moisture content percentage. The group C and D products were characterized by the highest moisture contents. In the cheeses supplied by producers A and B, the moisture content was considerably lower, being 56.84% on average. In the present study, the pH analysis revealed differences (*p* ≤ 0.05) between the ricottas supplied by producers B and D (Table 2). Acidity was lowest in the group B cheeses (pH = 5.63) and highest in the group D cheeses (pH = 6.19). No differences in acidity were observed between the group A and C ricottas, where the pH reached 5.89 on average. The data presented in Table 2 show that the ricottas supplied by producers C and D were characterized by similar titratable acidity levels (8 and 9 °SH, respectively), and significantly (*p* ≤ 0.01) different (and the highest) titratable acidity was noted in the group A and B cheeses (19 and 15 °SH, respectively).

The shear force values of the examined ricottas (Table 2) indicate that the group A cheeses were less brittle and harder (4.60 N) than the cheeses supplied by producers B, C, and D, which were characterized by significantly (*p* ≤ 0.01) lower shear force values (by 2.81, 3.52, and 3.06 N, respectively). The ricottas supplied by producer C were the most brittle ones (1.08 N), and they were characterized by a uniform structure and consistency, high spreadability, and an absence of clumps.

### 3.2. Color Parameters of Ricotta Cheeses

The color parameters measured on the surfaces of the analyzed ricottas are presented in Table 3. The cheeses supplied by producers A and D were characterized by significantly (*p* ≤ 0.01) higher values for lightness (L*) (93.33 and 92.76, respectively) than the group B and C ricottas. The examined ricottas differed (*p* ≤ 0.01) in the values of parameter a*, denoting the contribution of greenness (negative values) and redness (positive values), and parameter b*, denoting the contribution of blueness (negative values) and yellowness (positive values). Parameter a* assumed negative values (from −0.18 to −0.29) on the surfaces of the group C and D cheeses, which points to a shift towards a green color. In turn, positive values for parameter b* (7.78 to 15.95) were observed in all experimental groups. The color on the surfaces of the group B ricottas was characterized by the highest contribution of red and yellow components (a* = 2.65, b* = 15.95) (Table 3). The lowest contribution of yellowness was observed on the surfaces of the group C cheeses (b* = 7.78). The analyzed ricottas differed (*p* ≤ 0.01) in color saturation (chroma) due to differences in the values of parameters a* and b*. The chroma value was highest on the surfaces of the group B cheeses (C* = 16.17) and lowest on the surfaces of the group C ricottas (C* = 7.78). The color measured on the surfaces of the group C and D ricottas was characterized by the significantly (*p* ≤ 0.01) lowest negative values for the hue angle (h° = −88.69 and −88.38, respectively) relative to the cheeses purchased from producers A and B. The color measured on the surfaces of the group A cheeses was characterized by the highest positive value for the hue angle (h° = 87.48), which corresponded with the highest value of L* in this group. The value of h° on the surfaces of the group B cheeses was also positive but significantly (*p* ≤ 0.01) lower (by 6.93) than that for group A. The data presented in Table 3 show that the WI measured on the surfaces of the studied cheeses was significantly (*p* ≤ 0.01) the highest in the group C ricottas (WI = 88.90), relative to the ricottas supplied by producers D, A, and B. Color indicators and attributes are modified by non-enzymatic browning reactions, including the Maillard reaction, degradation of ascorbic acid, lipid peroxidation, and caramelization [40]. The YI is also determined in analyses of milk color to provide information about browning reactions [41]. In the present study, the examined ricottas differed (*p* ≤ 0.01) in their YI values. The YI was highest in the group B cheeses (25.06), which were also characterized by the lowest WI and h° values. In turn, the lowest YI value was found in the group C ricottas (12.06 on average).

Significant (*p* ≤ 0.01) differences in parameter L* were noted at the cross-sections of the analyzed cheeses (Table 3). Lightness values measured at the cross-section were highest in the group C (L* = 94.62) and group A (L* = 94.03) ricottas. The analyzed parameter was considerably lower at the cross-section of the group B cheeses. The examined samples also differed (*p* ≤ 0.01) in the contributions of redness and yellowness at the cross-section. The contribution of the red component was highest in the group of cheeses supplied by producer B (a* = 2.31), whereas the lowest negative value of a*, signifying a shift towards a green color, was noted in the group D cheeses (a* = −0.42). The highest contribution of yellowness was observed in the ricottas supplied by producer B (b* = 14.53). Parameter b* was lower by 1.26, 5.98, and 4.32 in the group A, C, and D cheeses, respectively. The obtained data (Table 3) point to significant (*p* ≤ 0.01) differences in chroma and hue angle at the cross-sections of the analyzed ricottas. Parameter C* was highest for the cheeses supplied by producer B (14.53) and lowest for those supplied by producer C (8.55). The hue angle assumed positive values for the ricottas supplied by producers A and B (87.45 and 80.96, respectively), and it was negative for the group D ricottas (−87.65). The WI and YI values determined at the cross-sections of the studied cheeses are presented in Table 3. The WI was significantly (*p* ≤ 0.01) the highest in the group C ricottas (89.89) relative to the cheeses supplied by other producers. The WI value was significantly lowest at the cross-sections of the group B ricottas (82.85). The cheeses supplied by producer B were characterized by the significantly (*p* ≤ 0.01) highest YI value (22.59) relative to the remaining experimental groups. The lowest YI values were noted for the group C and D cheeses. High YI values could point to a higher rate for the aging process.

### 3.3. Sensory Quality of Ricotta Cheeses

**Figure 2:**
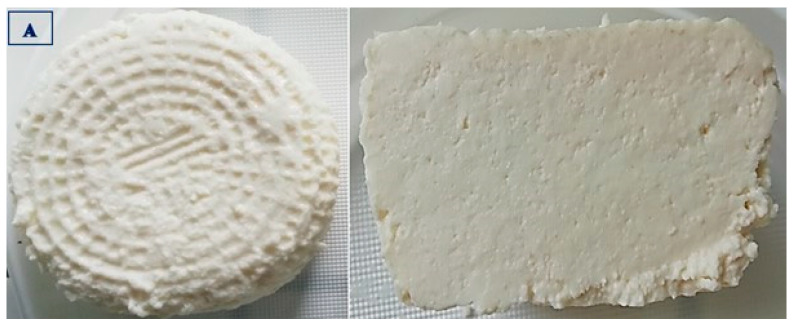
Ricotta cheese manufactured by producer A: external and cross-section appearance.

**Figure 3:**
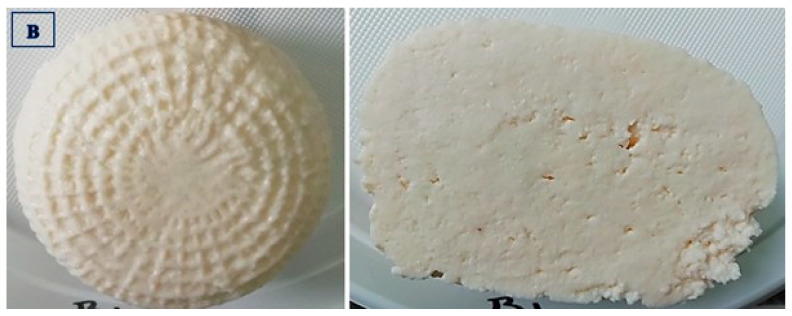
Ricotta cheese manufactured by producer B: external and cross-section appearance.

**Figure 4:**
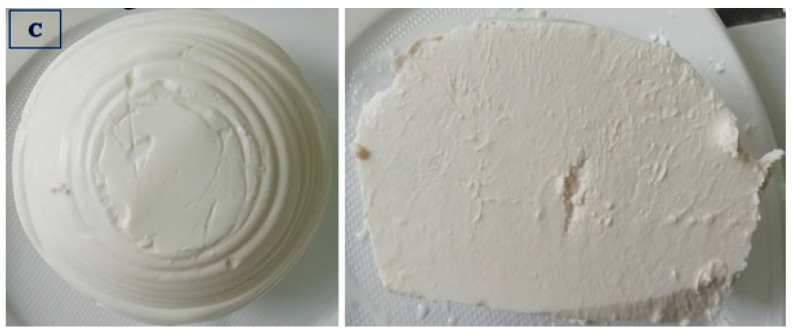
Ricotta cheese manufactured by producer C: external and cross-section appearance.

**Figure 5:**
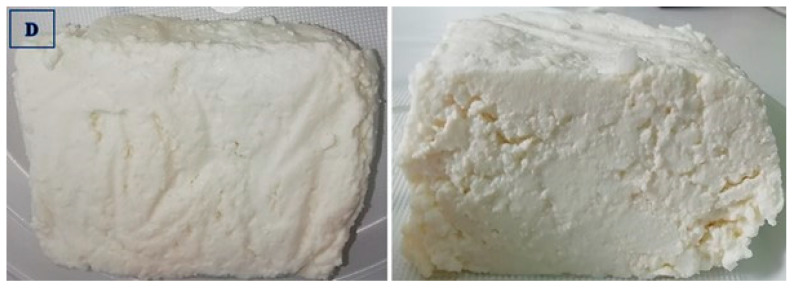
Ricotta cheese manufactured by producer D: external and cross-section appearance.

The data presented in Table 4 show that the ricottas purchased from producer B differed (*p* ≤ 0.01) in color from the remaining experimental groups. In the sensory evaluation, the cheeses supplied by producers A, C, and B received the highest scores for the most desirable (white to light cream) and uniform color, which testifies to the high quality of these products. The group D cheeses received the lowest score, whereas the group A, C, and B cheeses received the highest scores for appearance at the cross-section, and the noted differences were statistically significant (*p* ≤ 0.01) (Figure 2, Figure 3, Figure 4 and Figure 5).

The group D cheeses were characterized by visible spaces between grains and numerous cracks, which could be attributed to their high moisture content (72.57%) and a relatively low fat content declared by the producer (11 g/100 g of the product). The most uniform, cohesive, and grainy structure and consistency were noted in the ricottas supplied by producers A (4.75 points) and B (4.27 points). However, the group C ricottas received the highest score (4.86 points), whereas the group D cheeses received the lowest score (2.52 points) for structure and consistency, and the difference between these groups was statistically significant (*p* ≤ 0.01). It should be noted that only the ricottas supplied by producer C were homogenized and therefore characterized by a smooth and spreadable consistency and the absence of a typical grainy structure. In turn, the group D cheeses had a brittle and grainy structure, as well as a somewhat sticky consistency with clearly visible whey exudation. The data presented in Table 4 show that the group C ricottas differed (*p* ≤ 0.01) from the other experimental cheeses in terms of taste and aroma and received the lowest scores for these sensory attributes (3.55 and 3.47, respectively). The taste and aroma of the cheeses supplied by producer C deviated from the parameters that are typical of ricotta, and they were characterized by an intense foreign and impure aroma and aftertaste with a distinctive flavor of artificial additives. In terms of aroma and taste, the panelists gave the highest scores to the group A (4.69 and 4.67 points, respectively) and group D (4.41 and 4.47 points, respectively) ricottas. The aroma and taste of these products was described as pure and pungent but also delicate and creamy with a discernible sweet aftertaste.

## 4. Discussion

Giangolini et al. [42] examined the chemical composition of ricotta Romana cheeses made from sheep’s milk whey and determined their moisture content to be 70.59%. In turn, in the work of Souza et al. [43], the moisture content of 30 samples of ricotta cheeses supplied by five different producers reached 58.4% on average and was lower than that in the present study. Based on Brazilian standards relating to the moisture content percentage of cheeses, one sample in the above study was characterized by the average moisture content, and one sample—with high moisture content and 28 samples—was characterized by an extremely high moisture content. Souza et al. [43] concluded that most ricotta cheeses available in Belo Horizonte had normal moisture contents. In a study by Sulieman et al. [44], the moisture content of ricotta cheeses produced in a laboratory from whey purchased from a local market in Sudan was determined to be 72.89%. In the work of Borba et al. [45], the moisture content of ricotta made from a mixture of goat’s milk and cow’s milk whey as well as whole goat’s milk and cow’s milk ranged from 74.59% to 73.81% during 14 days of refrigerated storage. Ortiz Araque et al. [46] determined the moisture contents of full-fat (3.0%), low-fat (1.5%), and fat-free (<0.1%) ricotta cheeses to be 52%, 53%, and 57%, respectively. According to Streiff et al. [47], ricotta cheeses manufactured from whey at different production yields were characterized by moisture contents in the range of 69.75–79.24%. In the work of Kowalik et al. [16], the average moisture content of ricotta was 62.9% in cheeses produced from goat’s milk whey, 76% in cheeses made from cow’s milk whey, and 81.63% in cheeses manufactured from cow’s milk whey (95%) and milk (5%). In a study by Tripaldi et al. [48], the average moisture content of ricotta made from buffalo milk whey reached 65.51%, and it was similar to that reported by Mucchetti and Neviani [3] in Ricotta di Bufala Campana cheeses (64.58%). Jasińska and Skryplonek [5] observed clear differences in the moisture contents of three ricotta cheeses produced from cow’s milk whey and one cheese made from buffalo milk whey, where the analyzed parameter ranged from 67.8% to 85.5%. In a study by Madalozzo et al. [17], the moisture content of ricotta cheeses available in Brazil also varied considerably from 60% to 81%. Jasińska and Skryplonek [5] reported that Tasty Ricotta and Ricottina cheeses with the same declared composition did not differ in moisture content (82.77–85.50%, which was highest in the group of the four compared cheese variants). According to Jelen [49], the moisture content of unripened ricotta cheeses should range from 68% to 82%. The moisture contents of Ricotta Fresca and Ricotta Di Bufala analyzed by Jasińska and Skryplonek [5] were determined to be within the above range. The moisture content of Ricotta Fresca ranged from 73.93% to 77.50% and was most similar that that noted by El-Sheikh et al. [13] in ricotta manufactured from ultrafiltered whey (75.95%). In turn, Ricotta Di Bufala had a lower moisture content that ranged from 67.82% to 70.29% and was most similar that that reported by Pizzillo et al. [4] in ricotta produced from goat’s milk whey (68–71%). The moisture content of ricottas made from cow’s milk whey and acidified with vinegar, lemon juice, or grapefruit juice ranged from 67% to 79% [12]. In the cited study, the analyzed parameter gradually decreased during 60 days of refrigerated storage. According to Jasińska and Skryplonek [5], the fat content of ricotta is correlated with its moisture content and tends to increase with the loss of moisture. In the current study, only the group C and D cheeses were characterized by moisture contents that are typical of ricotta, remaining within the range of values indicated by Jelen [49].

In the work of Siemianowski et al. [8], the average pH of ricottas supplied by four producers ranged from 5.88 to 6.01. Esper et al. [50] also reported considerable differences in the chemical composition and acidity of ricottas available on the Brazilian market. In the cited study, the pH levels of 15 ricotta brands ranged from 4.95 to 6.26. According to Kowalik et al. [16], the pH level of fresh ricottas exceeds 6.0. The pH level of the ricottas examined by Sulieman et al. [44] was 5.3, whereas Ortiz Araque et al. [46] found that the acidity of ricotta cheeses ranged from 5.6 to 5.8 and was influenced by the fat content. In the cited study, ricottas made from milk with the addition of calcium chloride had a higher pH (6.2). Salvatore et al. [7] reported much higher pH values (6.63–6.79) in ricottas produced from sheep’s milk whey with different protein concentrations. In turn, Tripaldi et al. [48] found that the pH level of ricottas produced from buffalo milk whey decreased from 6.90 to 6.55 during 21 days of storage in the original packaging at a temperature of 4 °C. The decrease in the pH of stored cheeses can be attributed to the growth of lactic acid bacteria [3]. In the work of Mancuso et al. [51], the pH of fresh traditional sheep ricotta packaged in a modified atmosphere decreased from 6.54 to 5.97 on storage day 21. In turn, Modler [52] observed that the pH of ricotta made from a blend of whey and milk (80:20) ranged from 5.6 to 5.8, whereas the pH of ricotta produced from ultrafiltered whey only (from 4.5 to 1) was 5.7–5.9. Ricottas produced from the whey of four goat breeds were also characterized by a high pH, ranging from 6.27 to 6.43 [4]. According to Jasińska and Skryplonek [5], the pH of ricotta cheeses on the day of purchase ranged from 6.34 (Ricotta Fresca) to 6.57 (Ricottina). In addition, the active acidity and titratable acidity changed during refrigerated storage. The pH of Tasty Ricotta was highest (6.67) after 3 days of storage and lowest (5.96) after 10 days of storage. Ricotta Di Bufala was characterized by the highest pH (6.70) after 3 days of storage and the lowest pH (6.09) after 10 days of storage. In the cited study, the pH levels of cheeses supplied by four producers were similar to that reported by El-Sheikh et al. [13] in ricottas made from ultrafiltered whey (5.91), cheeses acidified with citric acid (5.96–6.21), and cheeses with the addition of gluconolactone (5.61–5.90). Borba et al. [45] found that the pH levels of ricottas increased significantly from 6.77 to 6.86 after 14 days of storage at a temperature of 7 °C. In the work of Di Pierro et al. [53], the pH of ricotta decreased in the first days of storage (from 6.63 to 6.39), which was attributed to the activity of lactic acid bacteria, and then the pH value stabilized and increased in successive days, reaching 6.47 in the fourth week of storage. Martins et al. [54] also reported an increase in the pH of ricotta in the fourth week of storage. The observed increase in pH could be due to microbial degradation of proteins, indicating the onset of product spoilage. The cited authors concluded that the high pH and high moisture content of ricotta cheeses shortens their shelf life, even when these products are stored under refrigerated conditions.

In the work of Jasińska and Skryplonek [5], the titratable acidity on the day of purchase was 6 and 7 °SH in Ricottina and Tasty Ricotta, respectively, and 10 and 11 °SH in Ricotta Fresca and Ricotta Di Bufala, respectively. In the cited study, ricottas made from cow’s milk whey were characterized by the highest titratable acidity (11 °SH) after 10 days of refrigerated storage (i.e., 1 day before the expiry date). In ricottas produced in a laboratory from whey purchased from a local market in Sudan, the titratable acidity (percent of lactic acid) reached 1.38 and was 0.35% higher than the value recommended by the DTU National Food Institute [55]. Di Pierro et al. [53] reported that the titratable acidity (percent of lactic acid) of ricottas increased in the first two weeks of refrigerated storage and remained constant during the remaining storage period (weeks 3 and 4). These changes were attributed to a gradual decrease in the activity of bacteria of the genus *Lactobacillus*, which produce lactic acid as the main metabolic end product, as well as amino acids and free fatty acids during proteolysis and lipolysis. In a study by Abdel-Razig and AlGamry [12], the activity of lactic acid bacteria induced an increase in the titratable acidity of stored ricottas acidified with vinegar, lemon juice, and grapefruit juice. The titratable acidity also increased during the refrigerated storage of tvorog, which is a fresh, unripened cheese with a short shelf life [56]. A decrease in the titratable acidity of ricottas at the end of their shelf life is indicative of alkalization and may be due to the development of undesirable microflora and product spoilage [54].

In the study by Ortiz Araque et al. [46], the hardness of ricottas with different fat contents was measured directly after production and was determined to be 1.7 N in full-fat cheeses, 2.2 N in low-fat cheeses, and 5.2 N in fat-free cheeses. On the 10th and last day of refrigerated storage in polypropylene packaging, the hardness increased to 2.6 N, 4.1 N, and 5.4 N in the above groups, respectively. In the cited study, the hardness of cheeses produced with the addition of four different coagulants (acetic acid, citric acid, lactic acid, and calcium chloride) was low (1.35 N and 1.25 N on storage days 0 and 10, respectively). Siemianowski et al. [8] demonstrated that the ricotta supplied by producer D, made from pasteurized cow’s milk whey, sweet cream, salt, and lactic acid, was characterized by a significantly (*p* ≤ 0.05) higher hardness (4.58 versus 2.73–2.09 N), higher gumminess (1.98 versus 1.19–1.42 N), and lower adhesiveness (−9.60 versus from −15.09 to −16.72 N·s) than the cheeses supplied by producers A, B, and C. Similar hardness values on the day of purchase (2.32–5.68 N) were reported by Jasińska and Skryplonek [5] for ricotta cheeses supplied by four producers in the Polish market. In the cited study, the analyzed cheeses differed significantly in hardness. According to Borba et al. [45], the addition of skim milk decreased the content of fat in cheese dry matter and increased the hardness, gumminess, and elasticity of ricotta, whereas the addition of whole milk increased the percentage of fat in the cheese dry matter and improved the texture of the ricotta. In the cited study, the hardness values of the ricotta cheeses were similar on storage days 1 and 14 (1.95 and 1.93 N, respectively).

Colorimetric measurements provide important information about the quality of dairy products and changes in quality parameters during storage. The color of dairy products is increasingly often measured to directly monitor the production process. The results are used to optimize each stage of production and to ensure that all technological processes take place under the appropriate conditions [57]. The color of dairy products is largely influenced by their composition, and this can change during processing and storage [58]. Siemianowski et al. [8] reported significant differences in the color lightness of ricottas supplied by four producers. The average value of parameter L* was 85.02 and was considerably lower than in the current study. In the cited work, all examined ricottas were characterized by negative values for parameter a* (from −0.65 to −1.12), which points to a shift towards a green color. In turn, the average values of parameters b* and C* were 7.47 and 7.53, respectively. Similarly to the present study, Borba et al. [45] found that some ricottas produced from a blend of goat’s and cows’ milk whey, whole goat milk, and whole cow’s milk were characterized by negative values for a* and positive values for b*. However, the cited authors reported a lower value for L* (78.59) on the first day of storage, a greater shift towards a green color (a* = −3.12), and a low value for parameter b* (8.59). After 14 days of refrigerated storage, parameter L* increased to 93.84, and the contribution of yellowness increased from 8.59 to 10.93. Borba et al. [45] also observed a decrease in the intensity of the green color (a* = −2.81). In the work of Pizzillo et al. [4], parameters a* and b* in ricottas made from the milk of four goat breeds assumed positive values in the range of 1.19–1.32 and 7.65–8.13, respectively. In the cited work, lightness values ranged from 93.63 to 94.31 and were similar to those noted in the current study. Ortiz Araque et al. [46] found that full-fat (3.0%), low-fat (1.5%), and fat-free (<0.1%) ricottas were characterized by similar values for L* (89.00) and a* (−3.26) directly after production. In turn, the contribution of yellowness was significantly influenced by the fat content of the ricotta. Parameter b* was highest (19.30) in the full-fat cheeses, and it was determined to be 12.30 in fat-free cheeses and 17.10 in low-fat cheeses. In the cited study, ricottas produced with the use of four different acidity regulators were characterized by similar values for L*, a*, and b*, which were determined to be 91.77, −3.10, and 15.87, respectively, on average.

Product quality is also described as consumer acceptance, and this important indicator should be regularly monitored and controlled. Sensory analysis is a helpful and subjective tool for evaluating the attributes of sensory attractiveness, such as color, taste, aroma, and texture. The sensory attributes of food products change in each stage of production, storage, and distribution, which is why the conditions during these processes should be monitored [59]. Fresh ricotta should have a cohesive and grainy structure with a delicate and pasty consistency, a milky or creamy aroma, a slightly sweet taste, and a high moisture content [3,28]. According to Kolanowski [26], the slightly sweet taste of ricotta can be attributed to the presence of lactose. In the first stage of sensory analysis, cheeses are evaluated for external appearance, rather than their taste, aroma, or appearance at the cross-section. Initially, similar to mozzarella, the delicate, white, and glossy surface undergoes changes induced by water evaporation and enzymatic reactions [60]. In a study by El-Sheikh et al. [13], ricottas produced using different methods received various scores on a 100-point scale in sensory analysis. Ricottas made from ultrafiltered Edam cheese whey without the addition of skim milk powder (SMP) received higher scores for appearance (9.2 on a 10-point scale), taste (36.6 on a 40-point scale), and consistency and texture (46.6 on a 50-point scale) than ricottas fortified with 2%, 4%, and 6% SMP. The control group products had a soft, moist, and grainy consistency, and their sensory quality was evaluated as being good. In addition, ricotta made from Edam cheese whey and acidified with 0.14 g/kg of citric acid received higher scores than ricotta acidified with 1.5% gluconolactone. Ricottas with the highest content of SMP received lower scores for the quality assessment. In turn, the cheeses fortified with 2% and 4% SMP had a sweet taste. El-Sheikh et al. [13] demonstrated that the addition of SMP (up to 2%) to Edam cheese whey was sufficient to obtain ricotta cheese with quality comparable to control group cheeses without added SMP. In the work of Ortiz Araque et al. [46], full-fat and low-fat (1.5%) ricottas received higher scores for color, aroma, texture, and general appearance (6.7, 6.05, 6.05, and 6.3 points on a 9-point scale, respectively). In turn, the fat-free (<0.1%) ricotta received lower scores for color (5.3 points), aroma (3.9 points), texture (3.4 points), and overall appearance (4.0 points). In an experiment conducted by Jasińska and Skryplonek [5], Ricotta Di Bufala made from buffalo milk whey received the lowest scores for aroma (3.5 points) and taste (3.5 points). The Ricotta Di Bufala had an intense aroma and a taste typical of buffalo milk, and as the only homogenized product, it had a smooth appearance without the grainy structure characteristic of ricotta made from cow’s milk whey. The Ricotta Di Bufala was characterized by the highest fat content, and its consistency was described as greasy and creamy. In the work of Jasińska and Skryplonek [5], Tasty Ricotta and Ricottina received similar scores for all sensory attributes. The Tasty Ricotta received slightly higher scores for its distinctive aroma (five points) and taste (five points). Both cheeses had a delicate and creamy aroma and taste, with a distinctive sweet aftertaste. Tasty Ricotta and Ricottina received 5.0 and 4.5 points, respectively, for a consistency that was described as grainy, delicate, soft, and somewhat pasty, with increasing levels of whey exudation on successive days of storage. However, the Ricottina was characterized by a more delicate structure and greater whey exudation. In the group of cheeses made from cow’s milk whey, Ricotta Fresca received the lowest score due to its dry and crumbly consistency (4.5 points), as well as its bland aroma (4.0 points) and taste (4.0 points). The Ricotta Fresca had a white color, while the Tasty Ricotta and Ricottina were characterized by a creamy white color, whereas the Ricotta Di Bufala had a white color with a grayish hue.

## 5. Conclusions

In the present study, the ricottas supplied by producers C and D were characterized by the highest moisture content and the lowest titratable acidity and shear force. The acidity (pH) was highest in the group B cheeses. Significant differences in color parameters measured on the surfaces and at the cross-sections were observed between the examined groups of cheeses. The ricottas supplied by producer A were characterized by the highest values of lightness on the surface, whereas the group B cheeses were characterized by the highest contribution of redness and yellowness, as well as the highest color saturation (chroma). The highest WI and YI values measured on the surface were determined to be for the cheeses supplied by producers C and B, respectively. Color lightness at the cross-section was highest in the group A and C ricottas. The contributions of redness and yellowness, chroma, and YI values were highest at the cross-sections of the group B cheeses. In turn, the group D ricottas were characterized by the greatest shift towards a green color at the cross-section, and the WI value at the cross-section was highest in the group C products. The cheeses supplied by producers A, C, and D were characterized by the most desirable color, which was evaluated as excellent. The group C ricottas received the lowest scores for aroma and taste, which were described as satisfactory. The examined ricottas also differed significantly in appearance at the cross-section, structure, and consistency. These quality attributes received the lowest scores in the cheeses supplied by producer D, which were characterized by visible spaces between grains, cracks, and a brittle, crumbly consistency.

In addition to differences in the organoleptic and physicochemical characteristics of the ricotta cheeses available in shops, it has been noted that producers do not indicate on the label the type of milk used to produce the ricotta, which is extremely important from the consumer’s point of view. The evaluation of the quality of ricotta cheeses available on the dairy products market should be continuously analyzed and monitored in terms of nutritional value, fatty acid profile, and microbiological quality due to the various raw materials used (i.e., milk from a cow, goat, sheep, or buffalo or milk whey), the different production methods, and the conditions of cold storage of these products.

## Figures and Tables

**Table 1:** Ingredient composition and nutritional and energy value of ricotta cheeses according to label data, as declared by manufacturers.

Specification	Producer			
	A (per 100 g)	B (per 100 g)	C (per 100 g)	D (per 100 g)
Composition	Pasteurized milk whey (80%), pasteurized milk (19%), pasteurized sweet cream, salt, acidity regulator: citric acid	Milk whey, pasteurized milk, salt, acidity regulator: citric acid	Milk whey, salt, acidity regulator: citric acid	Milk whey, salt, acidity regulator: citric acid
Net weight	250 g	220 g	250 g	240 g
Fat, including saturated fatty acids	22 g 15 g	14 g 9.8 g	9.5 g 6.7 g	11 g 7.3 g
Protein	11 g	12 g	8.4 g	9.7 g
Carbohydrates, including sugars	3.7 g 3.2 g	3.6 g 3.0 g	4.3 g 3.8 g	3.3 g 3.3 g
Salt	0.30 g	0.50 g	0.32 g	0.30 g
Energy value	1051 kJ/254 kcal	783 kJ/188 kcal	567 kJ/136 kcal	628 kJ/151 kcal
Storage temperature	1–8 °C	1–8 °C	0–4 °C	2–6 °C

**Table 2:** Analysis of selected physicochemical parameters of ricotta cheeses (mean ± SD).

Parameter	Producer				*p* Value
	A (n = 10)	B (n = 10)	C (n = 10)	D (n = 10)	
Moisture content (%)	55.38 ^D^ ±0.50	58.30 ^C^ ±0.32	73.52 ^A^ ±0.24	72.57 ^B^ ±0.65	0.001
pH	5.83 ±0.39	5.63 ^b^ ±0.05	5.96 ±0.28	6.19 ^a^ ±0.16	0.050
Titratable acidity (°SH)	19.00 ^A^ ±0.82	15.00 ^B^ ±1.83	8.00 ^C^ ±0.01	9.00 ^C^ ±0.82	0.001
Shear force (N)	4.60 ^A^ ±0.46	1.79 ^B^ ±0.13	1.08 ^C^ ±0.03	1.54 ^BC^ ±0.36	0.001

^A–D^: Mean values within a row marked with different uppercase letters differ significantly at *p* ≤ 0.01. ^a,b^: Mean values within a row marked with different lowercase letters differ significantly at *p* ≤ 0.05.

**Table 3:** Analysis of the color parameters of the ricotta cheeses (mean ± SD).

Parameter	Producer				*p* Value
	A (n = 10)	B (n = 10)	C (n = 10)	D (n = 10)	
On the Surface					
L*—lightness	93.33 ^A^ ±0.22	90.93 ^C^ ±0.64	92.14 ^B^ ±0.67	92.76 ^AB^ ±0.81	0.001
a*—redness	0.63 ^B^ ±0.05	2.65 ^A^ ±0.04	−0.18 ^C^ ±0.06	−0.29 ^D^ ±0.06	0.001
b*—yellowness	14.43 ^B^ ±0.68	15.95 ^A^ ±0.20	7.78 ^C^ ±0.17	10.37 ^D^ ±0.77	0.001
WI—whiteness index	84.08 ^C^ ±0.56	81.45 ^D^ ±0.24	88.90 ^A^ ±0.96	87.34 ^B^ ±0.98	0.001
YI—yellowness index	22.09 ^B^ ±1.00	25.06 ^A^ ±0.23	12.06 ^D^ ±0.27	15.97 ^C^ ±1.07	0.001
At the Cross-Section					
L*—lightness	94.03 ^AC^ ±0.26	91.85 ^B^ ±0.84	94.62 ^A^ ±0.43	93.40 ^C^ ±0.73	0.001
a*—redness	0.59 ^B^ ±0.03	2.31 ^A^ ±0.11	−0.06 ^C^ ±0.01	−0.42 ^D^ ±0.09	0.001
b*—yellowness	13.27 ^B^ ±0.18	14.53 ^A^ ±0.25	8.55 ^C^ ±0.10	10.21 ^D^ ±0.95	0.001
WI—whiteness index	85.44 ^B^ ±0.20	82.85 ^C^ ±0.79	89.89 ^A^ ±0.29	87.79 ^D^ ±1.04	0.001
YI—yellowness index	20.15 ^B^ ±0.29	22.59 ^A^ ±0.25	12.91 ^C^ ±0.19	15.62 ^D^ ±1.09	0.001

^A–D^: Mean values within a row marked with different uppercase letters differ significantly at *p* ≤ 0.01.

**Table 4:** Analysis of the sensory quality of the ricotta cheeses (mean ± SD).

Parameter (Point Score)	Producer				*p* Value
	A (n = 10)	B (n = 10)	C (n = 10)	D (n = 10)	
Color	4.72 ^A^ ±0.09	3.47 ^B^ ±0.30	4.86 ^A^ ±0.13	4.75 ^A^ ±0.18	0.001
External appearance at the cross-section	4.89 ^Aa^ ±0.18	4.08 ^Ab^ ±0.59	4.86 ^Aa^ ±0.20	3.08 ^B^ ±0.86	0.001
Structure and consistency	4.75 ^ACa^ ±0.14	4.27 ^Ab^ ±0.29	4.86 ^C^ ±0.07	2.52 ^B^ ±0.57	0.001
Aroma	4.69 ^Aa^ ±0.16	4.30 ^Ab^ ±0.34	3.55 ^B^ ±0.25	4.41 ^A^ ±0.23	0.001
Taste	4.67 ^A^ ±0.21	4.11 ^B^ ±0.25	3.47 ^C^ ±0.50	4.47 ^AB^ ±0.22	0.001

^A–C^: Mean values within a row marked with different uppercase letters differ significantly at *p* ≤ 0.01. ^a,b^: Mean values within a row marked with lowercase different letters differ significantly at *p* ≤ 0.05.

## Data Availability

The original contributions presented in the study are included in the article, further inquiries can be directed to the corresponding author.

## References

[1] Obrusiewicz T. (1995). Dairy Technology, Part 2.

[2] Chwastowska-Siwiecka I. (2021). Ricotta cheese-production, characteristics and quality features. Food Ind..

[3] Mucchetti G., Neviani E. (2006). Microbiologia e Tecnologia Lattiero-Casearia.

[4] Pizzillo M., Claps S., Cifuni G.F., Fedele V., Rubino R. (2005). Effect of goat breed on the sensory, chemical and nutritional characteristics of ricotta cheese. Livest. Prod. Sci..

[5] Jasińska M., Skryplonek K. (2015). Characterisctic of selected quality features of ricotta cheeses during refrigerated storage. Pol. J. Comm. Sci..

[6] Mangione G., Caccamo M., Natalello A., Licitra G. (2023). Graduate Student Literature Review: History, technologies of production, and characteristics of ricotta cheese. J. Dairy Sci..

[7] Salvatore E., Pes M., Falchi G., Pagnozzi D., Furesi S., Fiori M., Roggio T., Addis M.F., Pirisi A. (2014). Effect of whey concentration on protein recovery in fresh ovine ricotta cheese. J. Dairy Sci..

[8] Siemianowski K., Mickiewicz D., Lis A., Tońska E. (2016). Rheological properties, texture and color of ricotta available on the Polish market. Acta Agroph..

[9] Kamel B., Boubaker K., Attia H. (2013). Implementation of ricotta cheese production process in Tunisia. Int. Food Res. J..

[10] Modler H.W., Emmons D.B. (2001). The use of continuous ricotta processing to reduce ingredient cost in ‘further processed’ cheese products. Int. Dairy J..

[11] Guatemim E.L.X., da Silveira S.M., Millezi A.F., Ferenz M., Costa K.D., Rossi P., Bampi G.B. (2016). Evaluation of the microbiological quality of ricotta cheese commercialized in Santa Catarina, Brazil. Food Sci. Technol..

[12] Abdel-Razig K.A., AlGamry A.S. (2009). Effect of natural acidifying agents and storage temperature on quality of unripend whey cheese. J. Sci. Technol..

[13] El-Sheikh M., Farrag A., Zaghloul A. (2010). Ricotta Cheese from Whey Protein Concentrate. J. Am. Sci..

[14] Żulewska J. (2018). Acid-heat coagulated cheese- alternative for whey. Pol. Dairy J..

[15] Maubois J.-L., Kosikowski F.V. (1978). Making ricotta cheese by ultrafiltration. J. Dairy Sci..

[16] Kowalik J., Łobacz A., Żulewska J., Rapacka A. (2019). Ricotta and mascarpone cheeses-production and food safety aspects. Pol. Dairy J..

[17] Madalozzo E.S., Sauer E., Nagata N. (2015). Determination of fat, protein and moisture in ricotta cheese by near infrared spectroscopy and multivariate calibration. J. Food Sci. Technol..

[18] Fedele V. (2001). Grazing for different quality of cheeses. Caseus Int..

[19] Morand-Fehr P., Sanz Sampelayo M.R., Fedele Y.V., Le Frileux Y., Eknaes M., Schmidely P., Giger-Reverdin S., Bas P., Rubino R., Havrevoll O. Effects of feeding on the quality of goat milk and cheeses. Session 5: Nutrition and feeding strategies. Proceedings of the 7th International Conference on Goats.

[20] Rubino R., Moioli B., Fedele V., Pizzillo M., Morand-Fehr P. (1995). Milk production of goats grazing native pasture under different supplementation regimes in southern Italy. Small Rumin. Res..

[21] Agabriel C., Coulon J.B., Journal C., Sibra C., Albouy H. (1999). Variabilité des caractéristiques des fromages saint-nectaire fermiers: Relations avec la composition du lait et les conditions de production. Le Lait.

[22] Ferlay A., Martin B., Pradel P., Coulon J.B., Chilliard Y. (2006). Influence of grass-based diets on milk fatty acid composition and milk lipolytic system in Tarentaise and Montbeliarde cow breeds. J. Dairy Sci..

[23] Romanzin A., Corazzin M., Piasentier E., Bovolenta S. (2013). Effect of rearing system (mountain pasture vs. indoor) of Simmental cows on milk composition and Montasio cheese characteristics. J. Dairy Res..

[24] Smithers G.W. (2008). Whey and whey proteins- From ‘gutter-to-gold’. Int. Dairy J..

[25] Pintado M.E., Macedo A.C., Malcata F.X. (2001). Review: Technology, chemistry and microbiology of whey cheeses. Food Sci. Technol. Int..

[26] Kolanowski W. (2005). We serve cheese. Przegl. Gastr..

[27] Surażyński A., Nowak H., Kłobukowski J. (1977). „Ricotta”- type cheeses made from heat-acid coagulated milk. Pol. Dairy J..

[28] Kłobukowski J., Cichoń R. (2000). Nutritive value of some dairy products. Part II. Food Ind..

[29] (1973). Milk and Dairy Products-Cheeses-Methods of Analysis.

[30] Commission Internationale de L’éclairage (CIE) (1978). Recommendations on Uniform Color Spaces, Color-Difference Equations, Psychometric Color Terms.

[31] Rodriguez-Aguilera R., Oliveira J.C., Montanez J.C., Mahajan P.V. (2011). Effect of modified atmosphere packaging on quality factors and shelf-life of surface mould ripened cheese: Part I constant temperature. LWT-Food Sci. Technol..

[32] Rufián-Henares J.Á., Guerra-Hernandez E., García-Villanova B. (2006). Colour measurement as indicator for controlling the manufacture and storage of enteral formulas. Food Control.

[33] (2003). Sensory Analysis-Guidelines for the Use of Quantitative Response Scales.

[34] (2023). Sensory Analysis-Selection and Training of Sensory Assessors.

[35] (1991). Milk and Dairy Products-Unripened Curd Cheese.

[36] (1997). Milk and Dairy Products-Unripened Curd Cheese.

[37] (2017). Sensory Analysis-Methodology-General Guidance.

[38] Panfil-Kuncewicz H., Lis A., Majewska M. (2014). Effect of active packaging on microbiological shelf-life and sensory parameters of tvorog cheeses. Food Sci. Technol. Qual..

[39] StatSoft Inc. (2017). STATISTICA (Data Analysis Software System).

[40] Davies C.G.A., Labuza T.P. (1997). The Maillard Reaction Application to Confectionery Products.

[41] Francis F.J., Clydesdale F.M. (1975). Food Colorimetry: Theory and Applications.

[42] Giangolini G., Amatiste S., Filippetti F., Boselli C., Fagiolo A., Rosati R. (2009). Chemical composition of “Ricotta Romana” cheese. Sci. Tec. Latt.-Casearia..

[43] Souza M.R., Morais C.F.D., Corrêa C.E.S., Rodrigues R. (2000). Physico-chemical properties of Ricotta cheese marketed in Belo Horizonte, Minas Gerais. Rev. Hig. Aliment..

[44] Sulieman A.M.E., Eljack A.S., Salih Z.A. (2012). Quality Evaluation of “Ricotta” Cheese Produced at Laboratory Level. Int. J. Food Sci. Nutr. Eng..

[45] Borba K.K.S., Silva F.A., Madruga M.S., Queiroga R.C.R.E., Souza E.L., Magnani M. (2014). The effect of storage on nutritional, textural and sensory characteristics of creamy ricotta made from whey as well as cow’s milk and goat’s milk. Int. J. Food Sci. Tech..

[46] Ortiz Araque L.C., Darre M., Ortiz C.M., Massolo J.F., Vicente A.R. (2018). Quality and yield of Ricotta cheese as affected by milk fat content and coagulant type. Int. J. Dairy Technol..

[47] Streiff P.J., Nilson K.M., Dutwe A.H., Atherton H.V. (1979). Whey Ricotta Cheese Manufactured from Fluid and Condensed Whey. J. Food Prot..

[48] Tripaldi C., Rinaldi S., Palocci G., Di Giovanni S., Campagna M.C., Di Russo C., Zottola T. (2020). Chemical and microbiological characteristics of homogenised Ricotta Cheese produced from buffalo whey. Ital. J. Food Sci..

[49] Jelen P., Zadow J.G. (1992). Whey cheeses and beverages. Whey and Lactose Processing.

[50] Esper L.M.R., Bonets P.A., Kuaye A.Y. (2007). Avaliação das características físico-químicas de ricotas comercializadas no município de Campinas-SP e da conformidade das informações nutricionais declaradas nos rótulos. Rev. Inst. Adolfo Lutz.

[51] Mancuso I., Cardamone C., Fiorenza G., Macaluso G., Arcuri L., Miraglia V., Scatassa M.L. (2014). Sensory and microbiological evaluation of traditional ovine ricotta cheese in modified atmosphere packaging. Ital. J. Food Saf..

[52] Modler H.W. (1988). Development of a Continuous Process for the Production of Ricotta Cheese. J. Dairy Sci..

[53] Di Pierro P., Sorrentino A., Mariniello L., Giosafatto C.V.L. (2011). Chitosan/whey protein film as active coating to extend Ricotta cheese shelf-life. LWT-Food Sci. Technol..

[54] Martins J.T., Cerqueira M.A., Souza B.W.S., Do Carmo Avides M., Vicente A.A. (2010). Shelf Life Extension of Ricotta Cheese Using Coatings of Galactomannans from Non-conventional Sources Incorporating Nisin against *Listeria monocytogenes*. J. Agric. Food Chem..

[55] DTU (2009). National Food Institute: Cheese Whey.

[56] Karczewska D., Pikul J., Płuszka H., Chudy S. (2005). Changes in selected physicochemical characteristics of traditionally packaged tvarog in dependence on the type of packaging material used. Chłodnictwo.

[57] Dobrzańska A., Cais-Sokolińska D. (2014). Measuring the brightness and coordinate trichromaticity milk protein preparations. ABiD.

[58] Rój A., Przybyłowski P. (2012). Colour measurement of natural yoghurts. Bromat. Chem. Toksykol..

[59] Bzducha A., Obiedziński M.W. (2006). Instrumental methods for estimation of ripeness of fermented dairy products. Food Ind..

[60] McMahnon D.J., Fife R.L., Oberg C.J. (1999). Water Partitioning in Mozzarella Cheese and Its Relationship to Cheese Meltability. J. Dairy Sci..

